# Supplementary figures and images for: An α-Gal antigenic surrogate as a biomarker of treatment evaluation in Trypanosoma cruzi-infected children. A retrospective cohort study
Source: PLoS Negl Trop Dis. 2024 Jan 18;18(1):e0011910. doi: 10.1371/journal.pntd.0011910 (PMC10826959; doi:10.1371/journal.pntd.0011910)

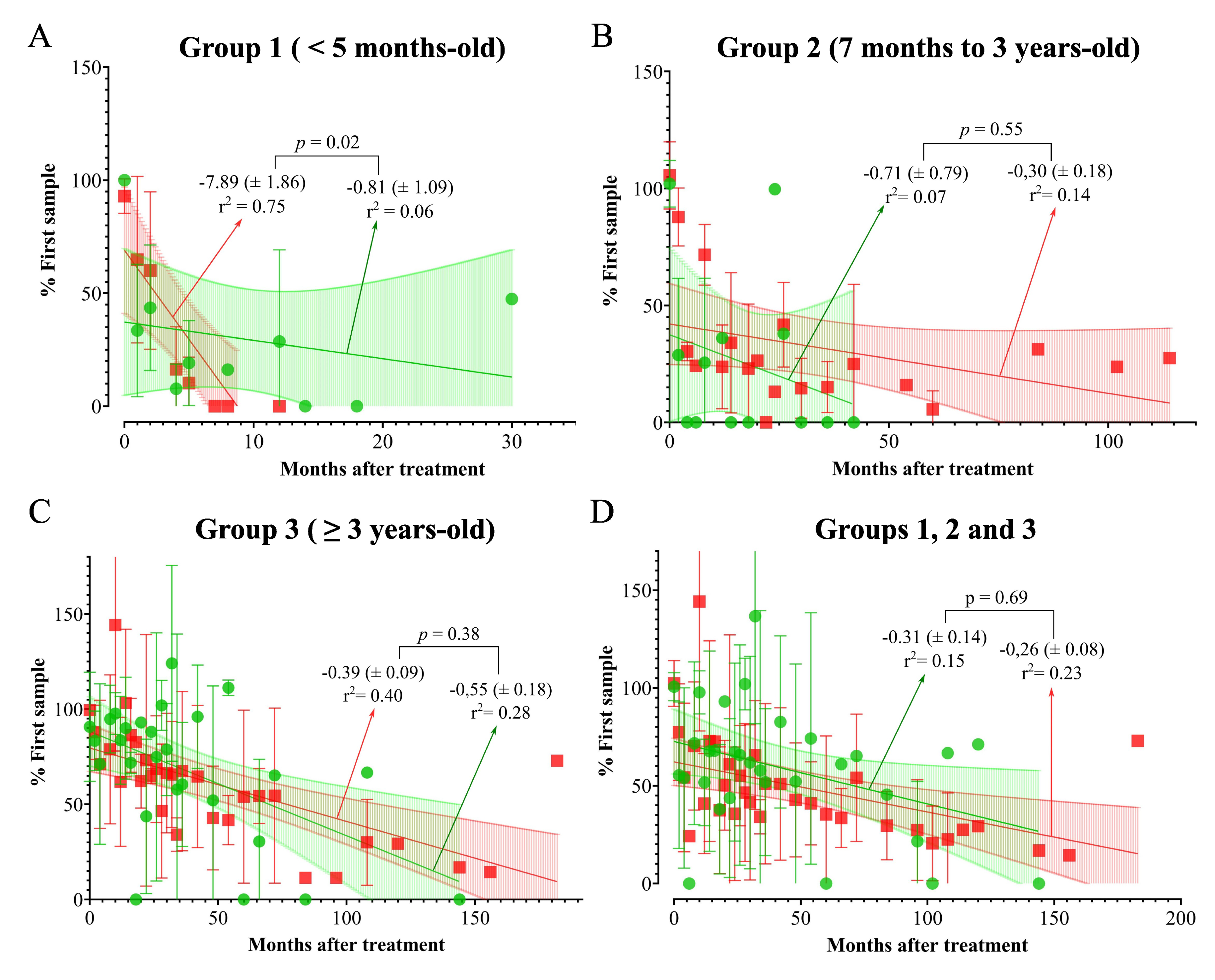

Supplement: S1 Fig — Serological profiles for tELISA and α-Gal-ELISA for patients from Group 1 (A), Group 2 (B), Group 3 (C) and the whole cohort of T. cruzi-infected children (D). Reactivity values are expressed as % of the first (pre-treatment, P) sample and regression curves are indicated in red and green lines, respectively. Mean reactivity and SD values for each time point are shown in red (tELISA) and green (α-Gal-ELISA) dots. Slope (95% CI) and R2 values are indicated for each data set. ANCOVA analyses were performed to compare slopes. (TIF) [file pntd.0011910.s003.tif]

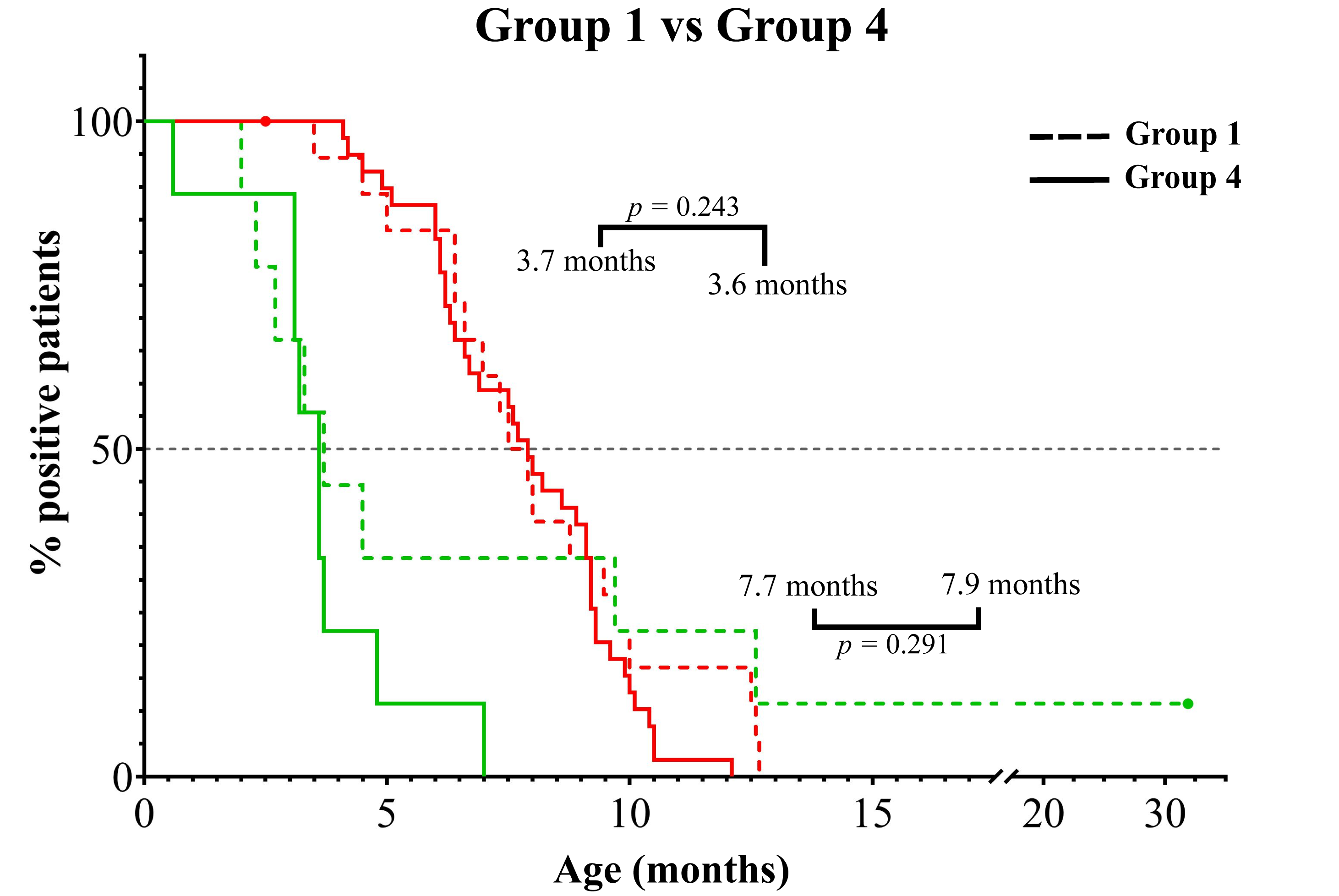

Supplement: S2 Fig — Kaplan-Meier curves showing the survival time for antibodies detected by tELISA (red) and α-Gal-ELISA (green) for patients from Group 1 (dashed lines) and Group 4 (solid lines). Median negativization values are indicated for each data set. Censored cases are indicated with dots. Log-rank (Mantel-Cox) analyses were performed to compare median time of negative seroconversion. (TIF) [file pntd.0011910.s004.tif]
