# Supplementary material for: An α-Gal antigenic surrogate as a biomarker of treatment evaluation in Trypanosoma cruzi-infected children. A retrospective cohort study
Source: PLoS Negl Trop Dis. 2024 Jan 18;18(1):e0011910. doi: 10.1371/journal.pntd.0011910 (PMC10826959; doi:10.1371/journal.pntd.0011910)
Supplement: Attachment — Submitted filename: Buscaglia and coworkers, PLOS ntd.pdf [file pntd.0011910.s005.pdf]

Buscaglia and coworkers describe the use of the synthetic neoglycoprotein BSA-Tri (consisting of BSA with multiple copies of the trisaccharide α-D-Galp(1→3)-β-D-Galp(1→4)-D-Glc conjugated to it), as a biomarker for chemotherapy efficacy when treating Chagas disease (CD) in babies and children. These patients were treated with benznidazole or nifurtimox, the only two drugs available for CD. Parasitological methods and PCR are not suitable to determine clearance of the parasite, which also hampers the development of new anti-CD drugs. The only acceptable method to determine cure from CD is negative antibody seroconversion, which requires a suitable antigen. *T. cruzi* trypomastigote lysates are not suitable for the serological determination of treatment efficacies due to the slow antibody seroconversion, which takes years. An alternative antigen is the *T. cruzi* derived F2/3 glycoprotein fraction, which is rich in terminal, highly immunogenic and antigenic α -Gal residues. The F2/3 can be used for the serological follow-up after treatment by monitoring a patients anti-alpha-Gal Ab levels, but the preparation of F2/3 from trypomastigotes is very challenging and not feasible on a large scale. Since currently no molecular carbohydrate biomarker is available, the authors used the above mentioned BSA-Tri for determining the treatment efficacy. Using α-D-Galp(1→3)-β-D-Galp(1→4)-D-Glc as a surrogate for F2/3 is a clever antigen choice because it contains the immunodominant glycotope α-D-Galp(1→3)-β-D-Galp, recognized by anti-alpha-Gal Abs of CD patients, and it is synthetically more accessible than the *T. cruzi* cell surface glycan α-D-Galp(1→3)-β-D-Galp(1→4)-D-GlcNAc. Before and after treatment, the authors measured the patients' anti-alpha-Gal levels by ELISA and found that seroconversion was significantly faster using BSA-Tri than conventional serology. Also, there was a trend that indicated that younger children seroconverted faster than older children. Despite the fact that the seroprevalence was rather low (only 58.4% of the children showed an Ab response to BSA-Tri) most likely due to sensitivity issues of the ELISA, this biomarker could proof useful in determining treatment efficacy in pediatric CD. Testing the performance of BSA-Tri is novel The manuscript is well written, the findings are significant and are of great interest to the readership of PLOS ntd. The In the opinion of this reviewer, the manuscript is suitable for publication in PLOS ntd after minor revisions.

Criticism:

1. The anomeric stereochemistry of the Glc units in BSA-Tri should be indicated. Is it alpha or beta?

2. Please comment on how it was determined that the second cohort, the babies of CD mothers, were indeed not infected with T. cruzi. If some of them were in reality *T. cruzi* positive, how will this affect data interpretation?

3. On page 6, please reference (a) Almeida et al., J. Clin. Lab Anal. 1993, DOI: 10.1002/jcla.1860070603; which demonstrates F2/3 as therapeutic marker in CD.

4. At the bottom of page 6, three references should be added, which suggest using T. cruzi-derived NGPs as prognostic biomarkers for chemotherapy follow-up:
(a) Ortega-Rodriguez, U. et al. *Methods Mol. Biol.* **2019**; doi: 10.1007/978-1-4939-9148-8_22.
(b) Montoya et al, Molecules 2022, https://doi.org/10.3390/molecules27175714. (This publication is referenced later in the manuscript, but should be referenced here already.)
(c) Alonso-Vega, C. et al., *BMJ Open*, **2021**, doi:10.1136/ bmjopen-2021-052897
