# Supplementary material for: An α-Gal antigenic surrogate as a biomarker of treatment evaluation in Trypanosoma cruzi-infected children. A retrospective cohort study
Source: PLoS Negl Trop Dis. 2024 Jan 18;18(1):e0011910. doi: 10.1371/journal.pntd.0011910 (PMC10826959; doi:10.1371/journal.pntd.0011910)
Supplement: Attachment — Submitted filename: Response to reviewers.docx [file pntd.0011910.s006.docx]

**Responses to Reviewers**

***Reviewer #1:*** The work is highly relevant for the development of new, improved diagnostic methods for the diagnosis of Chagas disease and evaluation of treatments' efficacy. The article deserves publication in PLOS Neglected Tropical Diseases.
**We kindly appreciate the reviewer’s comment.**

***Reviewer #2:*** Buscaglia and coworkers describe the use of the synthetic neoglycoprotein BSA-Tri (consisting of BSA with multiple copies of the trisaccharide α-D-Galp(1→3)-β-D-Galp(1→4)-D-Glc conjugated to it), as a biomarker for chemotherapy efficacy when treating Chagas disease (CD) in babies and children. These patients were treated with benznidazole or nifurtimox, the only two drugs available for CD. Parasitological methods and PCR are not suitable to determine clearance of the parasite, which also hampers the development of new anti-CD drugs. The only acceptable method to determine cure from CD is negative antibody seroconversion, which requires a suitable antigen. T. cruzi trypomastigote lysates are not suitable for the serological determination of treatment efficacies due to the slow antibody seroconversion, which takes years. An alternative antigen is the T. cruzi derived F2/3 glycoprotein fraction, which is rich in terminal, highly immunogenic and antigenic α -Gal residues. The F2/3 can be used for the serological follow-up after treatment by monitoring a patients anti-alpha-Gal Ab levels, but the preparation of F2/3 from trypomastigotes is very challenging and not feasible on a large scale. Since currently no molecular carbohydrate biomarker is available, the authors used the above mentioned BSA-Tri for determining the treatment efficacy. Using α-D-Galp(1→3)-β-D-Galp(1→4)-D-Glc as a surrogate for F2/3 is a clever antigen choice because it contains the immunodominant glycotope α-D-Galp(1→3)-β-D-Galp, recognized by anti-alpha-Gal Abs of CD patients, and it is synthetically more accessible than the T. cruzi cell surface glycan α-D-Galp(1→3)-β-D-Galp(1→4)-D-GlcNAc. Before and after treatment, the authors measured the patients' anti-alpha-Gal levels by ELISA and found that seroconversion was significantly faster using BSA-Tri than conventional serology. Also, there was a trend that indicated that younger children seroconverted faster than older children. Despite the fact that the seroprevalence was rather low (only 58.4% of the children showed an Ab response to BSA-Tri) most likely due to sensitivity issues of the ELISA, this biomarker could proof useful in determining treatment efficacy in pediatric CD. Testing the performance of BSA-Tri is novel The manuscript is well written, the findings are significant and are of great interest to the readership of PLOS ntd. The In the opinion of this reviewer, the manuscript is suitable for publication in PLOS ntd after minor revisions.

Criticism:

1. The anomeric stereochemistry of the Glc units in BSA-Tri should be indicated. Is it alpha or beta?

**Glc units are in beta anomeric stereochemistry. This point has been amended throughout the text, directly in the trisaccharide formula.**

2. Please comment on how it was determined that the second cohort, the babies of CD mothers, were indeed not infected with T. cruzi. If some of them were in reality T. cruzi positive, how will this affect data interpretation?

**As stated, this is a retrospective study. None of the patients in the second cohort was infected, as established by consistent negative results in serological analyses (using tELISA) at >8 months (see suppl Table 2). A statement clarifying this point has been added to the Methods section (subsection ‘Study Population and screening for T. cruzi infection’)**

3. On page 6, please reference (a) Almeida et al., J. Clin. Lab Anal. 1993, DOI: 10.1002/jcla.1860070603; which demonstrates F2/3 as therapeutic marker in CD.

**This reference has been added.**

4. At the bottom of page 6, three references should be added, which suggest using T. cruzi-derived NGPs as prognostic biomarkers for chemotherapy follow-up:
(a) Ortega-Rodriguez, U. et al. Methods Mol. Biol. 2019; doi: 10.1007/978-1-4939-9148-8_22.
(b) Montoya et al, Molecules 2022, https://doi.org/10.3390/molecules27175714. (This publication is referenced later in the manuscript, but should be referenced here already.)
(c) Alonso-Vega, C. et al., BMJ Open, 2021, doi:10.1136/ bmjopen-2021-052897

**These references have been added.**

***Reviewer #3:*** This is a relevant serological study aiming to define whether chagasic patients were cured or not by currently available drugs (benznidazol and nifurtimox). It is well established that conventional serology cannot distinguish patients that are chronically infected from those that were cured following chemotherapy. In past studies, two groups proclaimed that cured chagasic patients could be discriminated by measuring clearance rates of protective IgG antibodies directed against alfa galactose glycotope, either expressed in F2;3 surface antigens (authors previous work) or T. cruzi mucins (Almeida's group). Avoiding the complexity of antigen purification and batch variability of F2-3, here Buscaglia et al. screened sera from different age groups of pediatric cohorts (N=82 3-16 years old) using BSA coupled to a previously defined alfa-gal glycotope termed NGP-tri. The rate of clearance of IgG antibodies to NGP-tri was compared with antibodies directed to another well-characterized T. cruzi antigen (TSSA). Different curves were generated as a function of the children age, separated in 4 groups. Although the sensitivity of antibody detection in screens performed with NGP-tri is till not ideal for pediatric cases (the authors conceded that the technique can be improved), negative seroconversion was achieved in 23 months and 89.2% of the cases as compared to conventional serology (60 months, 43.2% of the patients). Moreover, the trend of negativization for NGP-tri was faster as compared to rates of TSSA specific antibodies.

Suggestions

Discussion
You could devote a paragraph to express your thoughts about the results obtained in "exceptional" cases. It is not clear why patient 1598 is PCR positive for T cruzi at 1.3 months and only treated at 19 months. Please address the obvious possibility that the early drop of alfa-Gal ELISA responses of this patient reflect competition of IgA serum antibodies (NGP-tri or TSSA) acquired during breast feeding. Microbiota is changing. Gradual elevation of serum IgA levels could even interfere with the early drop observed in 554 and 783, both treated with antiparasite drugs at time zero. From a perspective of basic science, this information (serum IgA level NGP-tri and TSSA antigens) would add interest to the work (readers of PLOS Neglected Trop Diseases have broader interests than clinical journals).

**Despite a PCR positive result, patient 1598 yielded negative results in hematocrit at 1.3 months and was therefore not treated. Unfortunately, PCR data could only be used to assist in Chagas disease clinics, but it has no diagnostic value.** **As for the possible rise of IgA responses towards alpha Gal, it is a very interesting observation. However, we are confident that this possibility could be ruled out because we used a peroxidase-conjugated goat IgG to human IgG/IgA/IgM immunoglobulins as secondary antibody**

References-There is an impressive number of self citations- please make sure that the proposition to use alga-gal glycopeptides from T. cruzi mucins as biomarkers of cure is properly cited both in the introduction and discussion. Making justice to the history of progress in each field is always important.

**We tried to be fair in the use of references, but the truth is that there are no many groups working on this issue (alfaGal and pediatric Chagas disease). In this revised version, we added a few citations suggested by other reviewers.**

Minor problems

Introduction
line 84- most important parasitic disease in Latin America- overstatement, please change the sentence- not justified anymore.

**This has been corrected**.

line 95-add reference after lower and variable.
**This has been corrected**.

line 98- their uncertain teratogenic risk. Suffice to write due to teratogenic risk
**This has been corrected**.

line 102- the only "accepted" criteria of cure is... - you must add a reference-either WHO or other health authority. Otherwise, change the sentence.

**This has been corrected**.

line 109. measure of parasite elimination - criteria would be better

**This has been changed as suggested by reviewer**.

line 126- inactivation of the alfa 1-3 galatosyltransferase gene (please describe the genetic change)

**This has been ammended**.

line 135- post therapeutic marker is not what you mean. Perhaps change for bker of cure...

**This has been corrected**.

Methods.
line 168-forgot to inform sex distribution

**See below.**

line 70. We are not informed how many were treated with BZN versus Nifurtimox

**Supplementary Tables 1 and 2 contain a complete list of all sera analyzed, as well as the sex and age and administered drug where this information was available.** 

line 211- be more precise- aforementioned is too economical
explain the criteria used to define group 4 (39 children) as non infected.

**This has been corrected**.
